# Identification of *piggyBac*-mediated insertions in *Plasmodium berghei* by next generation sequencing

**DOI:** 10.1186/1475-2875-12-287

**Published:** 2013-08-21

**Authors:** Yi Cao, Bing Rui, Dianne L Wellems, Mingxing Li, Biaobang Chen, Dongmei Zhang, Weiqing Pan

**Affiliations:** 1 Department of Tropical Infectious Diseases, Second Military Medical University, Shanghai 200433, China

**Keywords:** *PiggyBac* transposon, *Plasmodium*, Next generation sequencing

## Abstract

**Background:**

The *piggyBac* transposon system provides a powerful forward genetics tool to study gene function in *Plasmodium* parasites via random insertion mutagenesis and phenotypic screening. The identification of genotype of *piggyBac* mutants in the *Plasmodium* genome is thus an indispensable step in forward genetic analysis. Several PCR-based approaches have been used to identify the *piggyBac* insertion sites in *Plasmodium falciparum* and *Plasmodium berghei*, but all are tedious and inefficient. Next generation sequencing can produce large amounts of sequence data and is particularly suitable for genome-wide association studies. In this study, the Next generation sequencing technology was employed to efficiently identify *piggyBac* insertion sites in the genome of *P. berghei.*

**Methods:**

*Plasmodium berghei* parasites were co-transfected with *piggyBac* donor and helper plasmids. Initially, the classical inverse PCR method was used to identify the existence of *piggyBac* insertions in the *P. berghei* genome. The whole genome of post-transfection parasites was subsequently sequenced with a PCR-free paired-end module using the Illumina HiSeq sequencing system. The two distinct methods (‘BLAST method’ and ‘SOAP method’) were employed to identify *piggyBac* insertion sites in the *P. berghei* genome with Illumina sequencing data. All the identified *piggyBac* insertions were further tested by half-nested PCR.

**Results:**

The inverse PCR method resulted in a very low yield of ten individual insertions identified. Conversely, 47 *piggyBac* insertions were identified from about 1 Gb of Illumina sequencing data via the two distinct analysis methods. The majority of identified *piggyBac* insertions were confirmed by half-nested PCR. In addition, 1,850 single nucleotide polymorphisms were identified through alignment of the Illumina sequencing data of the *P. berghei* ANKA strain used in this study with the reference genome sequences.

**Conclusion:**

This study demonstrates that a high-throughput genome sequencing approach is an efficient tool for the identification of *piggyBac*-mediated insertions in *Plasmodium* parasites.

## Background

The completed and ongoing genome sequencing of several *Plasmodium* species has provided vast quantities of genomic sequencing data [1-4]. In addition, large-scale microarray, transcriptome and proteomic studies, and comparative genomic studies of different *Plasmodium* species and their life-cycle stages have generated substantial amounts of data on approximate 5,300 *Plasmodium* genes [5-8]. These studies provide valuable insights into the timing of expression and putative function of many encoded proteins. However, almost one-half of the predicted *Plasmodium* genes have no characterized orthologues outside the genus, and for most of these genes there exists no previous knowledge about their function. High-throughput novel approaches for dissecting and confirming the functions of *Plasmodium* genes are, therefore, needed. Although reverse genetics by targeted gene disruption or mutation is the most direct way to study gene functions, genome-scale and homologous recombination-based reverse genetics in *Plasmodium* is extremely challenging to perform due to very low transfection efficiency.

The forward genetic approach involving random insertion mutagenesis is now available in *Plasmodium falciparum* and *Plasmodium berghei* using *piggyBac* transposable elements derived from the cabbage looper moth, *Trichoplusia ni*[9-11]. The *piggyBac*-based transposon system, which has been widely used to manipulate the genomes of both invertebrate and vertebrate species [12-14], provides a powerful genetic tool to study the roles of genes through mass phenotypic screening of *Plasmodium* insertion mutants. *PiggyBac* transposon can be randomly inserted into the TTAA tetra-nucleotide sites of the *Plasmodium* genome generating a large number of insertions at different genomic loci. Hence, the identification of insertion sites in the *Plasmodium* genome is critical for the forward genetic analysis approach using *piggyBac* transposon mutagenesis. To date, several PCR-based approaches to detect insertion sites in *P. falciparum* and *P. berghei* have been reported [9,11]. However, all these approaches involve numerous PCR reactions, cloning and sequencing of PCR products, resulting in a tedious procedure that is not highly efficient for identification.

Next generation sequencing (NGS) is a revolutionary molecular biology research tool with a wide and rapidly growing range of applications, including genome *de novo* sequencing and resequencing, identification of genetic variants, detection and profiling of coding and non-coding transcripts, epigenetic profiling, and interaction mapping [15,16]. Using NGS data, several recent studies detected the presence or absence of transposon copies in the genomes of several model species by different computational methods [17,18]. In this study, the Illumina HiSeq system was applied to re-sequence the whole genome of *P. berghei* ANKA parasites that was co-transfected with *piggyBac* donor and helper plasmids, in order to systematically identify the *piggyBac* insertion sites. In addition, the Illumina sequencing data was used to identify single nucleotide polymorphisms (SNPs) between the reference strain and the laboratory strain of *P. berghei* ANKA. *Plasmodium berghei* is the most frequently used model species for analysing *Plasmodium* gene function because it exhibits relatively high transfection efficiency and is easily manipulated throughout the complete life cycle, both *in vitro* and *in vivo*[19]. In this study, the analysis methods for the NGS data were established to identify *piggyBac* insertions more efficiently in the *P. berghei* genome. Dissection of gene function in *P. berghei* can be highly informative to malaria biology, because many genes are shared across *Plasmodium* species.

## Methods

### Plasmid construction

The donor and helper plasmids were constructed for *P. berghei* transfection. The pHTH plasmid and the minimal *piggyBac* plasmid pXL-BACII were generously provided by Dr Bharath Balu and Dr John H Adams [9]. The *piggyBac* transposase coding sequence was excised from the pHTH plasmid with BamHI and cloned into the BamHI site of the plasmid pL0006 (a gift from MR4) under the control of 5′ untranslated regions (UTR) of *P. berghei eef1aa* (*Pbeef1aa*) and 3′UTR of *P. berghei dhfr-ts* (*Pbdhfr-ts*), resulting in helper plasmid pL06PB for transient transposase expression.

**Figure 1:**
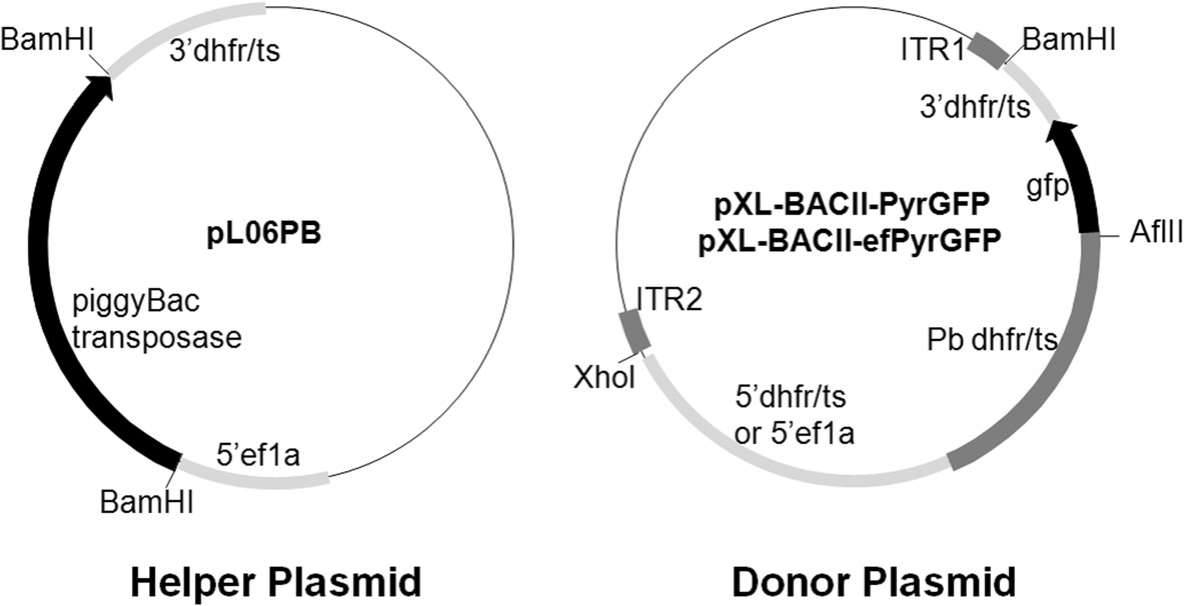
**The *piggyBac *helper plasmid and donor plasmids for co-transfection of *Plasmodium berghei *parasites.** The helper plasmid (left) contains the transposase gene under control of the constitutive *eef1aa* 5′UTR and the *dhfr/ts* 3′UTR for transient transposase expression. The two donor plasmids (right) contain the *Pb dhfr/ts* selectable marker and *gfp* fusion gene expression cassette under the control of constitutive *dhfr/ts* 5′UTR or *eef1aa* 5′UTR. The expression cassette is flanked by the *piggyBac* inverted terminal repeats (ITR).

The transposon donor plasmid pXL-BACII-PyrGFP was created by cloning a BamHI/Xhol fragment from the pPyrFlu plasmid (a gift from Dr Robert Ménard) into pXL-BACII plasmid. This fragment contained a PbDHFR-TS/GFP fusion protein expression cassette under the control of 5′UTR and 3′UTR of *Pbdhfr-ts*. Another transposon donor plasmid pXL-BACII-efPyrGFP was created by replacing the 5′UTR of *Pbdhfr-ts* in the plasmid pXL-BACII-PyrGFP with 5′UTR of *Pbeef1aa*. The 599-bp *Pbeef1aa* 5′UTR was amplified from plasmid pL0006 using primers ef5′F (5′-GATCTCGAGCCCAGCTTAATTCTTTTCAAGCTCTTTATGCTTA-3′) and ef5′R (5′-GATACGCGTCCCTATGTTTTATAAAATT-3′) and ligated into pMD18-T vector (Takara company, Japan), resulting in the intermediate plasmid. The 488-bp fragment from open reading frame of *Pbdhfr-ts* was amplified from plasmid pPyrFlu, using the primers PbDTF (5′-GATACGCGTATGGAAGACTTATCTGAAAC-3′) and PbDTR (5′-GGAAATAAATCATCTACAC-3′), and cloned into MluI/XbaI sites downstream of the *Pbeef1aa* 5′UTR in the intermediate plasmid. The Xhol /XbaI fragment was excised from the intermediate plasmid, and replaced the corresponding Xhol /XbaI fragment in the plasmid pXL-BACII-PyrGFP, generating transposon donor plasmid pXL-BACII-efPyrGFP (Figure 1).

### *Plasmodium berghei* co-transfection with transposon helper and donor plasmids

Transfection of *P. berghei* ANKA parasites were performed using the Amaxa Nucleofector system as described previously [19]. The parasites were simultaneously co-transfected with *piggyBac* helper plasmid (pL06PB) and either of two donor plasmids (pXL-BACII-PyrGFP and pXL-BACII-efPyrGFP). A total of ten co-transfection experiments were performed using a constant amount (2.5 μg) of donor plasmid per transfection. Among them, experiments with the ratios, 1:1, 2:1 and 4:1, of helper plasmid to pXL-BACII-efPyrGFP donor plasmid were performed three, two and three times, respectively, and experiments with the ratios, 1:1 and 2:1, of helper plasmid to pXL-BACII-PyrGFP donor plasmid were performed once respectively. As control *P. berghei* parasites were transfected with 2.5 μg of either of donor plasmids. Selection of resistant parasite populations after transfection was performed by treatment of the infected mice with pyrimethamine as described previously [19].

### Host leukocyte elimination and genomic DNA extraction

To eliminate the contamination of host genomic DNA, leukocytes in the mouse blood were removed using the commercial Plasmodipur filter [20]. Plasmodipur filters (Euro-Diagnostica, Arnhem, Netherlands) were prerinsed with sterile PBS solution. The whole blood samples were diluted with four volumes of PBS, then gently passed through the Plasmodipur filter using a syringe and collected in 50 ml tubes. Red blood cells (RBCs) remaining in the filter were washed with 10–15 ml of PBS solution and collected. The filtered RBCs were centrifuged at 800 g for 10 min. The supernatant was removed, and the pelleted erythrocytes were lysed in two volumes of 0.15% saponin/PBS for 30 min at 37°C. The released parasites were pelleted with 10,000 rpm for 10 min at 4°C, and washed three times with ice-cold PBS at 10,000 rpm for 10 min at 4°C. The parasite pellets were stored at −20°C for DNA extraction. The genomic DNA was extracted from parasite pellets using QIAamp DNA Blood Midi Kit (Qiagen company, Germany) as per manufacturer’s protocol. The quality of the extracted genomic DNAs was examined by Nanodrop and agarose gel electrophoresis.

### Inverse PCR method for identification of insertion sites in the genome

The location of *piggyBac* insertions in the *P. berghei* genome was initially identified by the inverse PCR method. Two microgram of genomic DNA was digested for 4 hr in ten units of TaqI or AluI, precipitated with three volumes of ethanol and 1/10 volume of 3 M sodium acetate, and self-ligated overnight in 50 μl reaction. The ligation products were precipitated as above, resuspended in 20 μl distilled water. The TaqI-digested, self-ligated products were digested with ten units of TseI to remove the episomal fragments. The inverted terminal repeat (ITR) 2 insertion sites in TaqI-digested genomic DNA were identified to perform the inverse PCR with the six primers described previously in *P. falciparum*[9], and the ITR 2 insertion sites in AluI-digested genomic DNA were identified to perform the inverse PCR using the sense primers ( 5'-CGTCAATTTTACGCAGACTATC-3′; 5′-GAGAGCAATATTTCAAGAATG-3′ ) and the antisense primers ( 5′-CGAATCCGTCGCTGTG-3′; 5′-CATTTAGGACATCTCAGTCG-3′). The PCR products were cloned into pMD18-T vector and sequenced by using M13 forward and reverse primers. The insertion sites in the genome were determined by performing BLASTN search in the PlasmoDB database.

### Illumina sequencing of co-transfected *Plasmodium berghei* parasites

The DNA samples extracted from *P. berghei* parasites under transfection and drug selection were sequenced on the Illumina Hiseq2000 platform. Library preparation and sequencing was undertaken by the BGI preparation and sequencing teams (BGI, Beijing Genomics Institute at Shenzhen, Shenzhen, PRC). A PCR-free paired-end sequencing library with the 300 bp average length of DNA fragments (ranging from 200 to 400 bp) was prepared from ~10 μg total DNA using Illumina’s sample preparation kit, and sequenced with a paired-end module. The sequenced samples had 90 bp length of sequence reads.

### Detection of insertion sites with Illumina sequencing data

After filtering the raw reads with adapter sequences or low quality bases, the paired-end reads were mapped to the reference sequences of *P. berghei*[21] and *Mus musculus*[22] using the software of Short Oligonucleotide Alignment Program (SOAPaligner) [23]. The parameters while mapping were set as that the minimum and maximum insert sizes of paired-end reads were allowed respectively to 200 and 400 bp, and maximum two mismatches and no InDels were allowed. When one read was mapped to multilocations in the genome, only one locus was selected randomly. Only the sequences that did not map to the mouse reference sequences were included in the following analysis. Two different methods were employed to identify *piggyBac* insertion sites in the *P. berghei* genome with Illumina sequencing data.

### Method 1 (BLAST method)

**Figure 2:**
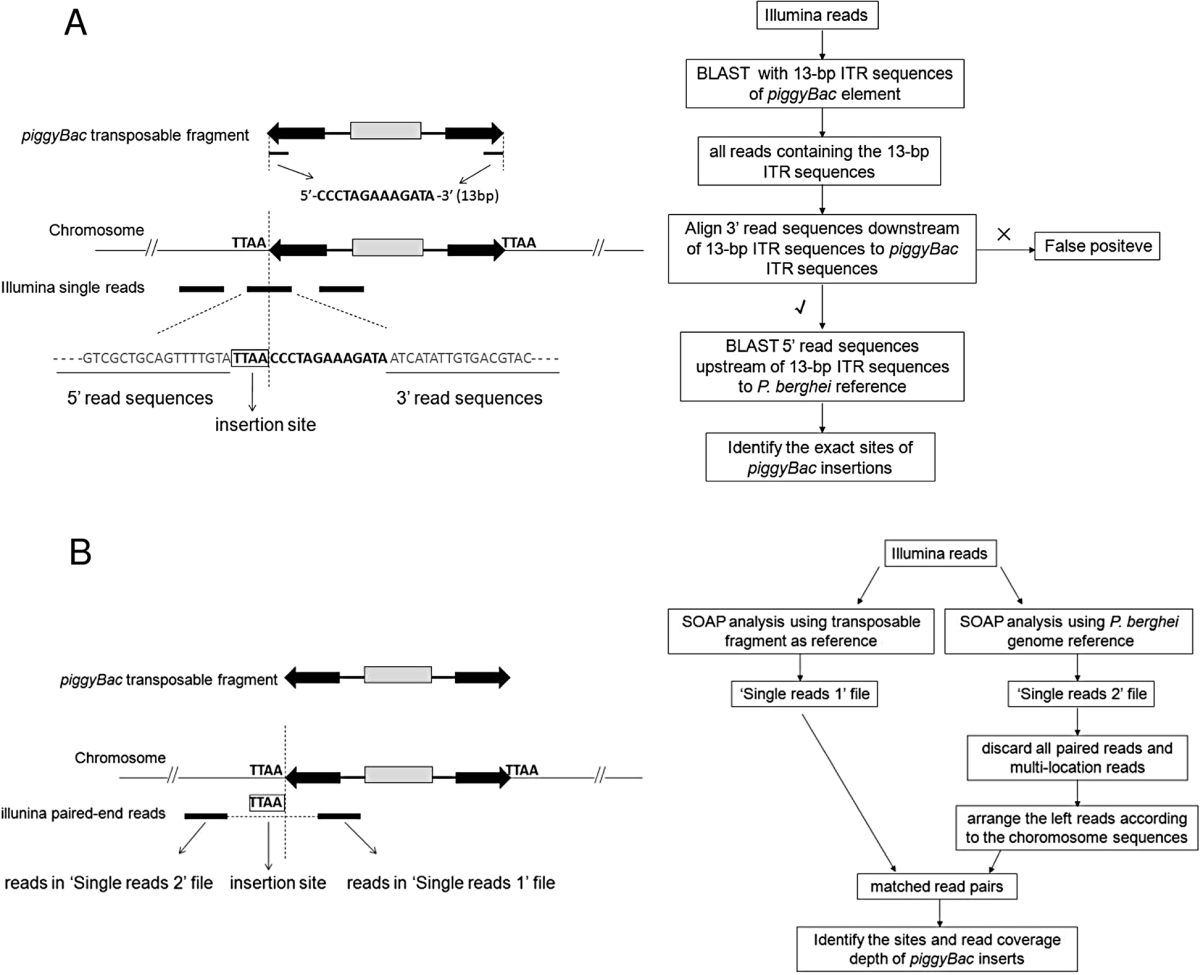
**Diagram depicting the two methods of detecting *piggyBac *insertion sites in *Plasmodium berghei *genome with Illumina sequencing data. (A)** The ‘BLAST method’ is based on BLAST searching with the 13-bp end sequences of *piggyBac* ITRs in the Illumina reads. All the Illumina sequencing reads were regarded as single-end reads. Each of single reads can be located in *P. berghei* chromosomes, or in the *piggyBac* transposable fragments, or at the junctions between the *piggyBac* inserts and the flanking genomic sequences. Only the reads overlapping the junctions can be used to identify the exact sites of *piggyBac* insertions. The example read comes from *piggyBac* insertion at 52719 of chromosome 1. The workflow of ‘BLAST method’ is shown at the right panel. **(B)** The ‘SOAP method’ is based on aligning the Illumina sequencing reads respectively to the *piggyBac* transposable fragments and *P. berghei* reference sequences using the SOAP software. The Illumina reads were sequenced with the paired-end module. Both of the paired-end reads can be located in the *P. berghei* chromosomes or in the *piggyBac* transposable fragments, or alternatively, one of the paired-end reads can lie in the *piggyBac* transposable fragments while the other of the paired-end reads can lie in the flanking genomic sequences. The latter can be used to identify the sites of *piggyBac* insertions. The workflow of ‘SOAP method’ is shown at the right panel.

The BLAST search was performed with the 13-bp end sequences (5′-CCCTAGAAAGATA-3′) of the ITRs of *piggyBac* element in all the read sequences after filtering the adapter sequences and low quality bases. Every read containing the 13-bp sequences was picked up from sequencing data, and then the read sequence was divided into two parts. The 5′ read sequences upstream of 13-bp end sequences were used as the query sequences for BLASTN against the *P. berghei* genome reference to determine the exact location of insertions in the genome. The 3′ downstream sequences were used to align against the known ITR sequences of *piggyBac* element to confirm the real transposition and avoid the false positive due to alignment of 13-bp end sequences in the genome reference (Figure 2A).

### Method 2 (SOAP method)

The SOAPaligner was used to align short reads generated by the Illumina sequencing onto appointed reference sequences. Firstly, the transposable fragment sequences from the donor plasmid were used as reference sequences, and then were aligned by the resequencing genome data. A large number of sequencing reads were identified and extracted into a ‘singles’ file, designed as ‘Single reads 1′. Secondly, all of the resequencing genome data were also aligned to the *P. berghei* reference sequences. Huge quantities of reads were mapped to the sequences of each chromosome, and extracted into another ‘singles’ file, designed as ‘Single reads 2′. Further processing to the file of ‘Single reads 2′ was performed by getting rid of all the paired reads and only keeping the single reads aligned to a specific locus in the *P. berghei* genome. The left single reads were arranged according to the reference sequences of each chromosome. Finally, the ID numbers of reads were matched between the ‘Single reads 1′ file and ‘Single reads 2′ file, and then the matched read pairs were found out. The positions of *piggyBac* insertions in the *P. berghei* reference genome were revealed by the positions of reads from the ‘Single reads 2′ file. The read depth was demonstrated by the number of the matched read pairs at the same genomic locus (Figure 2B).

### Experimental verification of insertion sites by PCR with site-specific primers

To experimentally verify the *piggyBac* insertion sites detected by Illumina sequencing and inverse PCR, the half-nested PCRs were performed for all identified insertion sites. All primers were designed using Primer5 software. Each insertion site was amplified by half-nested PCR with a site-specific primer (Additional file 1) and a pair of primers located in either of the *piggyBac* ITR arms (ITR1: 5′-CCTCGATATACAGACCGATAAAAC-3′ and 5′-GTTTGTTGAATTTATTATTAGTATGTAAGT-3′; ITR2: 5′-CTCCAAGCGGCGACTGAG-3′ and 5′-CATTGACAAGCACGCCTCAC-3′) using the genomic DNA of post-transfection parasites as templates. The PCR products were sequenced using the Sanger method with the corresponding primer located in *piggyBac* ITR arms. The resulting sequences were aligned to the *P. berghei* reference genome in the PlasmoDB database using BLASTN search. The exact sites of *piggyBac* insertions confirmed by the PCR products were compared with the predicted sites from the ‘BLAST method’, ‘SOAP method’ and inverse PCR method.

### Single nucleotide polymorphism analysis

To detect SNPs of *P. berghei*, the Burrows-Wheeler Aligner (BWA) software was used to map the Illumina sequencing reads to the reference genome of *P. berghei*[24]. In order to reduce the false positive rate, the reads with InDel and the duplication reads were removed. Then SOAPsnp [25] was used to assemble the consensus sequences based on the alignment reads. The SNPs were identified on the consensus sequences through comparison with the reference. The final SNPs set was passed through the following filtering criteria: at least 5 bp between two neighbouring SNPs; the approximate copy number of flanking sequence was less than two; the quality value was more than 20; and the depth of supported reads at that locus was no less than three.

## Results

### Transfection of *Plasmodium berghei* ANKA parasites

*Plasmodium berghei* parasites were co-transfected with *piggyBac* donor plasmid and helper plasmids simultaneously (Figure 1). Without a drug-selection cassette the helper plasmid, pL06PB, could transiently express the *piggyBac* transposase in the transfected parasites under the control of the constitutive promoter from the *Pbeef1aa* gene. Two donor plasmids contained the 5′ and 3′ ITRs of the *piggyBac* element, which were the minimal cis-elements necessary for *piggyBac* mobilization. A fusion protein expression cassette of drug selectable marker and gfp was located inside two ITRs under the control of *Pbdhfr-ts* 5′UTR or *Pbeef1a* 5′UTR. Therefore, transfection of the donor plasmids would result in insertion of this fusion protein expression cassette into the TTAA target sites in the *P. berghei* genome. The expression of the drug selectable marker and gfp fusion protein allowed selection of parasites by pyrimethamine and detection of parasites with green fluorescence. The 5′UTR of *Pbdhfr-ts* and *Pbeef1a* could constitutively express the fusion gene in the blood stage of parasite. The co-transfections of parasites were performed at various ratios of the helper plasmid and the donor plasmids, and the drug-selected parasites showed green fluorescence.

### Identification of insertion sites by inverse PCR method

Initially, a standard inverse PCR method (iPCR) was used to identify the location of *piggyBac* insertions in the *P. berghei* genome. To increase the possibility of finding insertion sites, the genomic DNA extracted from post-transfection parasites was digested by TaqI and AluI, respectively [26]. The digested self-ligated fragments were used for amplification of the genomic sequences flanking the ITR2 element with the corresponding sense and antisense primers. Identification of insertions was confirmed by sequence analysis. Ten insertion sites were identified by the iPCR method (Additional file 1), indicating a very low yield for insertions identified by this method. Sequence analysis of these ten insertions confirmed a consensus TTAA-site-specific integration of the *piggyBac* transposable fragment into the parasite genome. No *piggyBac* insertion was found by inverse PCR in the control parasites transfected with either of donor plasmids.

### Whole genome Illumina sequencing of co-transfected *Plasmodium berghei* parasites

**Figure 3:**
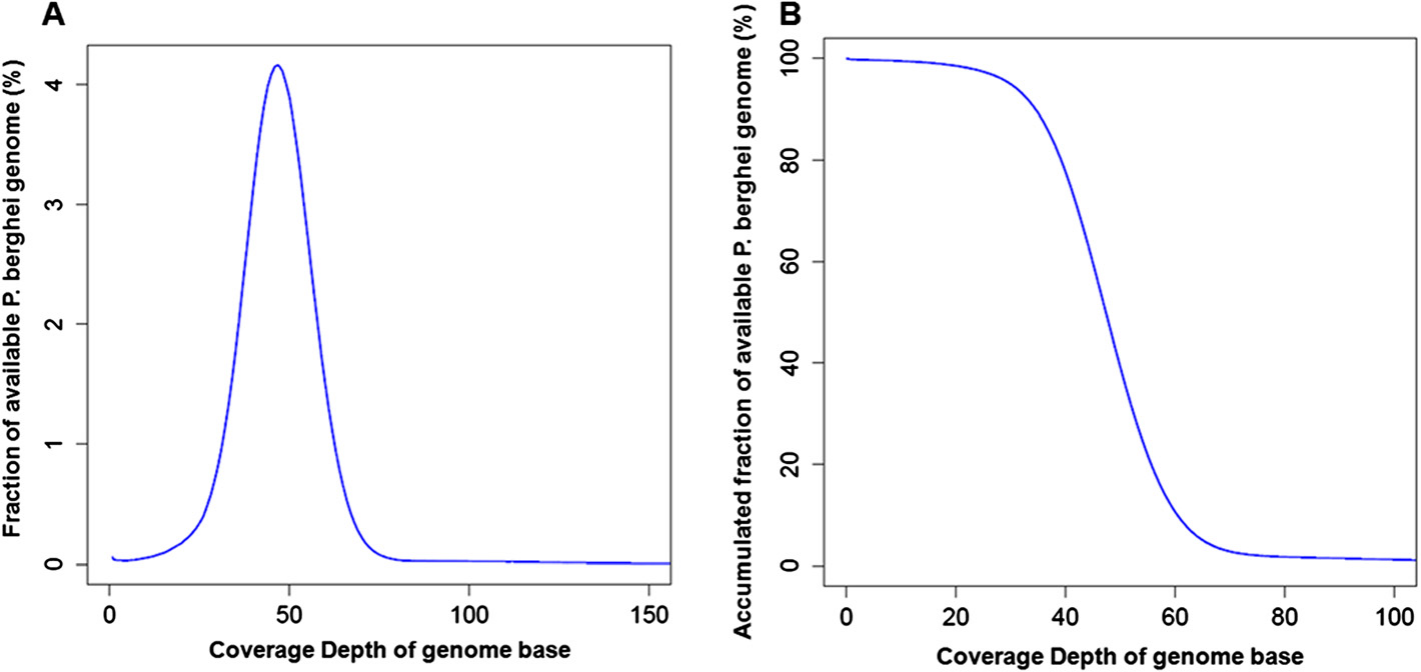
**Distribution of sequence coverage across the available reference genome of *Plasmodium berghei *ANKA. (A)** The percentage of the available genome plotted against the depth of genome base coverage. **(B)** The accumulated percentage of the available genome plotted against the depth of genome base coverage.

As the yield of insertions by iPCR method was low, the whole genome sequencing of the transfected parasites was applied to identification of insertions. Mixed genomic DNA extracted from the *P. berghei* parasites of ten co-transfection experiments were used to prepare the Illumina sequencing library. The whole genome sequencing produced 11,796,446 reads of 90 bp length, resulting in a total sequence pool of 1.06 Gb. The 0.879 Gb of the total sequence pool was mapped to the *P. berghei* genome, while 0.121 Gb of the total sequence pool was mapped to the mouse genome (Table 1). Removal of the host leukocytes improved the *P. berghei* sequencing yield by reducing mouse DNA levels to 11% (0.121 Gb in total 1.06 Gb) in the sequenced sample (Table 1). The sequences mapped to the mouse reference genome were not included in subsequent analyses. The coverage of sequenced data accounted for 99.8% of available *P. berghei* genome, and the coverage depth averaged 47.8 fold (Table 2). The Illumina data when mapped to the *P. berghei* genome showed a Poisson-like distribution with the peak observed around the average read depth. A plot of the accumulated fractions of available genome against the depth of base coverage revealed that 99.5% of genomic bases were covered by the mapped reads at ten times or greater, while 94.9% of genomic bases were covered at 30 times or greater (Figure 3). This finding indicates that the characteristic of these data was in line with that of the Illumina sequencing data from the PCR-free paired-end library, which had a better read distribution and more even genome coverage [27].

**Table 1:** The results of mapping the Illumina sequencing reads to the reference genome using the SOAPaligner program

**Reference genome**	**Total mapped reads**	**Total mapped bases (bp)**	**Mapping rate (%)**	**Mapped paired reads**	**Mapped single reads**
*P. berghei* ANKA	9,771,546	879,439,140	82.83	9,363,770	407,776
*Mus musculus*	1,343,876	120,948,840	11.39	1,163,314	180,562

**Table 2:** ***Plasmodium berghei *ANKA genome coverage by chromosome**

**Chromosome**	**Total base**	**Coverage base**	Coverage (%)*	**Even coverage depth**
	**(without N)**			
1	475,058	474,159	99.81	46.5
2	636,358	634,527	99.71	51.4
3	587,073	586,764	99.95	44.9
4	724,231	721,086	99.57	46.1
5	917,344	916,957	99.96	50.2
6	918,417	917,782	99.93	48.5
7	810,351	801,856	98.95	46.4
8	1,354,067	1,353,940	99.99	46.7
9	1,632,101	1,629,300	99.83	47.9
10	1,579,605	1,579,521	99.99	47.5
11	1,719,070	1,717,503	99.91	47.2
12	1,764,385	1,763,706	99.96	46.8
13	2,492,531	2,492,086	99.98	46.5
14	2,448,164	2,447,394	99.97	46.9
berg_bin	339,395	327,701	96.55	76.6
genome level	18,398,150	18,364,282	99.82	47.8

* % of nucleotides sequenced by ≥1 reads.

### Detection of insertion sites within Illumina sequencing data

Both the 5′ and 3′ ITRs of the *piggyBac* element consisted of a terminal 13 bp and internal 19 bp perfect inverted repeat separated by a 3 bp (5′ITR) or a 31 bp (3′ITR) spacer (Figure 2A). The blast search of 13-bp ITR sequence in the PlasmoDB failed to find perfect alignment in the published reference sequences of *P. berghei* and two other rodent malaria parasites (*Plasmodium yoelii* and *Plasmodium chabaudi*). To identify the insertion sites in the genome, BLAST search was performed with the 13-bp ITR sequence on all sequencing reads to identify the junction between the *P. berghei* genome and the transposable fragment in the read sequences. Total 21 insertion sites in the genome were identified via this ‘BLAST method’ (Additional file 1). All insertions were confirmed at the expected canonical “TTAA” site, which was just adjacent to the 5′ end of the 13-bp ITR sequence.

The paired-end Illumina data allowed to look for the insertion sites using the paired-end reads, in which one read was present inside the transposable fragment and the other read was in the *P. berghei* genome flanking the *piggyBac* insertion site (Figure 2B). A total of 29 genomic insertion sites were identified with the ‘SOAP method’ (Table 3). The majority (21) of these identified insertion sites were revealed by only a pair of reads, resulting in the insertion location in a range of 200–400 bp. The more paired reads identifying an insertion, the smaller range in which the insertion could be located. The two insertion sites identified with significantly higher read depth (20 paired reads or more) occurred at the loci of *P. berghei dhfr-ts* and *eef1aa*. Since the donor plasmids (pXL-BACII-PyrGFP and pXL-BACII-efPyrGFP) contained the endogenous fragments from *P. berghei dhfr-ts* and *eef1aa*, the higher read depth might not be due to the *piggyBac* insertion, but due to homologous recombination at the corresponding genomic sites via the endogenous fragments within two plasmids. (Table 3 and Additional file 1).

**Table 3:** The 29 genomic insertion sites identified by the ‘SOAP method’

**Chromosome**	**Range of insertion site**	**Number of supporting pair-end reads**
berg03	141655 ± (200 ~ 400 bp)	1
berg04	138106 ± (200 ~ 400 bp)	1
berg04	353862 ± (200 ~ 400 bp)	1
berg04	374523 ± (200 ~ 400 bp)	1
berg04	679800 ± (200 ~ 400 bp)	1
berg05	272425 ± (200 ~ 400 bp)	1
berg04	858235 ± (200 ~ 400 bp)	1
berg06	534191 ± (200 ~ 400 bp)	1
berg07	247428–247493 (65 bp)	6
berg07	653302–653595 (293 bp)	20
berg08	21190 ± (200 ~ 400 bp)	1
berg08	1143631 ± (200 ~ 400 bp)	1
berg08	1229311 ± (200 ~ 400 bp)	1
berg09	1385754 ± (200 ~ 400 bp)	1
berg10	859354 ± (200 ~ 400 bp)	1
berg11	396596–396660 (64 bp)	3
berg11	1132095 ± (200 ~ 400 bp)	1
berg11	1236263–1236275 (12 bp)	22
berg12	137149–137213 (64 bp)	4
berg12	1663662 ± (200 ~ 400 bp)	2
berg13	307464 ± (200 ~ 400 bp)	2
berg13	1325560 ± (200 ~ 400 bp)	1
berg13	1819639 ± (200 ~ 400 bp)	1
berg13	2109829 ± (200 ~ 400 bp)	1
berg13	2135273 ± (200 ~ 400 bp)	1
berg14	906574–906725 (151 bp)	3
berg14	190567 ± (200 ~ 400 bp)	1
berg14	1963049 ± (200 ~ 400 bp)	1
berg14	2223862 ± (200 ~ 400 bp)	1

### PCR verification of insertion sites

**Figure 4:**
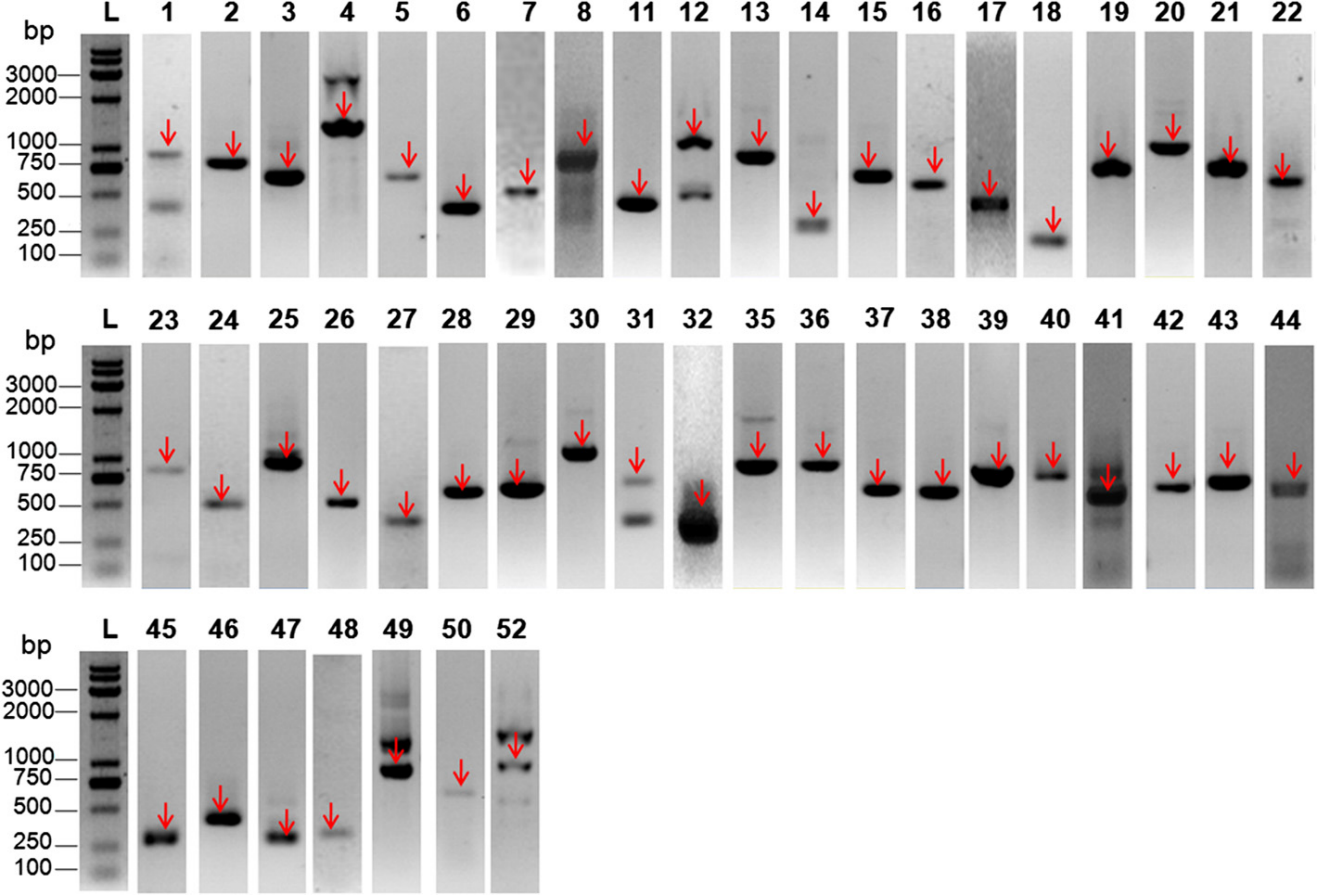
**PCR amplification of all insertion sites identified by the ‘BLAST method’, the ‘SOAP method’ and the ‘iPCR method’.** Half-nested PCR products were amplified by each of site-specific primers and a pair of primers located in either of the *piggyBac* ITR arms using the genomic DNA of post-transfection parasites as templates. The number of each lane coincides with the number shown in Additional file 1; L = DNA ladder.

The reliability of identified insertion sites by the ‘BLAST method’, the ‘SOAP method’ and the iPCR method was tested by half-nested PCR amplification of all *piggyBac* insertions in the post-transfection parasites. All the 21 insertion sites identified by the ‘BLAST method’ except one generated PCR products of expected sizes. The exact sites of the insertions verified by blasting the sequences of PCR products in PlasmoDB agreed with the sites previously identified by the ‘BLAST method’. Nine of the ten insertion sites identified by the iPCR method generated the expected PCR products, and blasting the sequences of PCR products in PlasmoDB also verified these sites previously identified by iPCR method. Of the 29 insertion sites identified by the ‘SOAP method’, 25 obtained the specific half-nested PCR products, while the remaining four sites obtained no-specific PCR products. The exact sites of these 25 insertions were confirmed by aligning the sequences of PCR products to the *P. berghei* reference genome and fell in the genome range predicted by the ‘SOAP method’. In total, 47 insertion sites were confirmed by sequencing of PCR products (Figure 4 and Additional file 1).

### Analysis of *Plasmodium berghei* single nucleotide polymorphism

Pair-ended PCR-free reads were aligned to the *P. berghei* reference genome using BWA software, and 1,850 SNPs were identified using SOAPsnp, the majority of which were heterozygous (>90%, 1,709 SNPs; Table 4). Of these 1,850 SNPs, 334 (18.05%) were mapped to the *P. berghei* “bin”, a composite “chromosome” with 0.339 Mb (1.84% of genome sequences available) from the contigs that were not located to any specific chromosome. Since, the “bin” sequences often came from the genome regions with low complexity and repetitive sequences yielding inferior reference sequence quality, the ambiguity of read mapping could increase and lead to the false positive and false negative in SNP calling. These 334 SNPs within *P. berghei* “bin” were discarded, and only the remaining 1,516 SNPs in the 14 chromosomes were analysed.

**Table 4:** **Distribution of the identified SNPs in the *Plasmodium berghei *ANKA genome**

**Chromosome**	**Homozygous**	**Heterozygous**	**Total**
	**SNPs**	**SNPs**	**SNPs**
berg01	12	11	23
berg02	5	196	201
berg03	1	22	23
berg04	9	141	150
berg05	11	36	47
berg06	1	66	67
berg07	17	114	131
berg08	3	107	110
berg09	10	73	83
berg10	3	71	74
berg11	6	151	157
berg12	4	156	160
berg13	8	91	99
berg14	5	186	191
berg_bin	46	288	334
SNPs in 14 chrs	95	1421	1516
All SNPs:	141	1709	1850

Among these 1,516 SNPs, 95 SNPs were homozygous and mostly covered by at least ten uniquely mapped reads (five SNPs were covered by eight uniquely mapped reads, and another four SNPs were covered by nine uniquely mapped reads, see Additional file 2). The remaining 1,421 SNPs in the chromosomes were heterozygous (Additional file 2). Of these heterozygous SNPs, 524 (34.6%) were identified under the more lenient criteria (the uniquely mapped reads supporting the newly detected base was less than ten and less than one-third of all the reads uniquely mapped to the same genomic site). This meant that each of these SNPs had fewer reads supporting the newly detected base, and simultaneously, had twice as many reads supporting the ‘reference base’. These SNPs were considered to be false positives, since the most likely bases at these sites were identical to the reference sequence (Additional file 2). Under the stricter criteria (the uniquely mapped reads supporting the newly detected base was at least ten and more than the uniquely mapped reads supporting the ‘reference base’), the 120 heterozygous SNPs were identified, which were considered as the truly heterozygous SNPs (Additional file 2).

## Discussion

The identification of insertion sites in the genome is a critical component of forward genetic analysis using transposon mutagenesis. Previous studies have described two PCR-based approaches to detect insertion sites in *P. falciparum* and *P. berghei*[9,11]. In the studies on *P. falciparum*, 177 unique *piggyBac* insertions were identified using the classical iPCR method in 81 independent transfections [9]. Since the number of insertions obtained in one transfection experiment with one million parasites was low, ranging from one to fourteen due to a low transfection efficiency (10^-6^-10^-8^), and parasite clones with various insertions could be obtained readily by limiting dilution methods *in vitro*, the classical iPCR was still viewed as a practical method to identify insertion sites in the *P. falciparum* genome [28].

Compared to *P. falciparum*, far higher transfection efficiency (10^-2^-10^-3^) has been achieved for *P. berghei* using AMAXA Nucleofection technology [19]. Studies have estimated that in parent populations obtained by drug selection of 1 × 10^6^-5 × 10^6^ transfected *P. berghei* parasites per transfection, 16 to 18 times more *piggyBac* insertions were generated compared with *P. falciparum* parasites [28]. However, *P. berghei* parasite cloning is usually performed by intravenous inoculation of a single parasite per mouse, which is more tedious and laborious than *P. falciparum* cloning. Hence, it is impractical to obtain all the clones from the parent population, and more *piggyBac* insertions need to be identified in the mixed post-transfection population.

In this study, attempts to identify *piggyBac* insertions by classical iPCR resulted in a very low yield of ten individual insertions. This finding is similar to that reported by Fonager *et al.*[11], indicating iPCR is not efficient for identifying *piggyBac* insertions in the transfected *P. berghei* population. Fonager *et al.*[11] also used the adapted TAIL-PCR method with semi-arbitrary degenerate primers to identify 127 insertions from two transfection experiments of *P. berghei* parasites. However, this method also involved in numerous PCR reactions, cloning and sequencing of PCR products [11].

NGS provides an ultrahigh throughput method with higher read depth and greater genome coverage. This has made NGS technologies suitable for genome-wide association studies, such as detecting SNPs and mapping mutations on a genome-wide scale. *PiggyBac* insertions produce mutations throughout the *Plasmodium* genome that generate novel sequence junctions that are absent in the reference sequence. This makes NGS technologies ideal for efficiently identifying *piggyBac* insertions.

In this study, the efficiency of two distinct methods was tested to detect the presence of individual insertions from Illumina sequencing data. First, when all the Illumina sequencing reads were regarded as single-end reads, the presence of a *piggyBac* insertion was indicated by the reads that overlapped the junction between a specific insert and its unique flanking genomic sequence. The sequences of these reads were chimeric of genomic sequence and transposable fragment sequence. In this method, the exact insertion sites were determined by blasting the flanking genomic sequences within the genome reference (except that the flanking sequences were repetitive or too short to be mapped into multiple genome sites, or that the reference was still unknown). Second, an alternative method that could be used only with paired-end sequencing reads was applied to identifying the *piggyBac* insertions. In this method, the presence of a *piggyBac* insertion was indicated by the paired-end reads, in which one read was present inside the transposable fragment and the other read was in the *P. berghei* genome region. One problem with this method was that each insertion site could only be positioned in a genomic location with a limited range. This is because the junctions between the transposable fragments and flanking genomic sequences were not present in both of the pair-ended reads. Therefore, the exact sites of these insertions were verified by additional PCR amplification with the site-specific primers and aligning the PCR sequences to the genome reference. Among all the identified insertion sites, only three were discovered by both of BLAST method and SOAP method. Due to distinct principles of the two methods, the probability of detecting a single insertion by both methods was small. Even when considering the insertion sites identified by the iPCR method, only six insertion sites were discovered by two or more methods. The parasites containing these six insertion sites might be dominant in the post-transfection population.

Due to the relatively low transfection efficiency, the majority of transformed *P. berghei* parasite clones were expected to have a single specific insertion in their genome. This suggests that each parasite clone with a specific insertion was only a small part of the multiclonal post-transfection populations. In one parasite clone the *piggyBac* insertion generated a novel sequence junction at its specific genomic locus, whereas in other parasite clones this locus was intact as the wild type. Even though a parasite clone could be relatively dominant in the mixed post-transfection population, its mutant genomic locus might still remain in the minority compared to the wild type of this locus. Thus, the coverage and read depth of NGS data had the decisive impact on the detection of *piggyBac* insertions. Forty-seven *piggyBac* insertions in the genome were detected in about 1G bp of the sequencing data by the BLAST method and SOAP method. As read depth increases with greater sequencing data, it is likely that more *piggyBac* insertions would be identified in the *P. berghei* genome. This might be particularly true for insertion sites in the non-dominant parasite clones of the mixed post-transfection populations. In both of BLAST method and SOAP method, the original sequencing reads were directly used, rather than the assembled contigs that were used in previous studies to detect the transposon insertions in *Drosophila melanogaster* strains[18]. Genomic DNA was randomly sheared into fragments for library construction, and hence at each genomic locus of *piggyBac* insertion the Illumina data contained both the reads from the wild type and from the insertion mutant. For this reason, assembling reads from any insertion mutant locus into the contigs was made difficult by the redundant reads from wild type of this locus.

Genetic diversity between the reference strain and *P. berghei* ANKA strain used in this study was assessed by examining single nucleotide polymorphisms. Although *piggyBac* insertions occurred at a number of genomic loci, they had no effect on SNP detection in the Illumina re-sequencing data. This is because the reads derived from exogenous sequences of *piggyBac* transposon element and the reads containing chimeric sequences from the *P. berghei* genome and *piggyBac* transposon element were discarded by the BWA software when calling SNPs. The results showed that of the 1,850 SNP passing the filtering criteria, only 95 homozygous SNPs were found in all 14 chromosomes, indicating that there was no significant genetic difference between *P. berghei* ANKA strain used in this study and the reference strain. These homozygous SNPs identified might be accumulated from mutations that occurred during the passage history of *P. berghei* ANKA line (for example, continuous asexual replication and growth rate selection). Another possibility is that some homozygous SNPs were present due to base errors in the published reference data [25].

Since malarial parasites in the blood stage are haploid, heterozygous SNPs identified in Illumina data are often considered to be derived from sequencing errors or from poor quality base calling [7]. In this study, heterozygous SNPs were dominant in the called SNPs, and even after passing filtering criteria the false positive rate in the called heterozygous SNPs remained high (524 of 1,421 heterozygous SNPs in 14 chromosomes). However, if assuming that ten or more read coverage was reliable for calling SNPs, it was found that 120 heterozygous SNPs were covered by at least ten uniquely mapped reads supporting the newly detected base, and at each of these SNP sites the reads supporting the newly detected base were in the majority of all mapped reads. It is likely that not all these SNPs originated from the Illumina sequencing errors. This is because the *P. berghei* ANKA parasites used in the transfections were not a clonal line, and the mixed parasite population was the main source of these heterozygous SNPs. Another possibility was that some heterozygous SNPs might be accumulated from mutations during the passage of the parasite line.

## Conclusion

This study has clearly demonstrated that high-throughput next generation genome sequencing is an efficient tool for the identification of *piggyBac*-mediated insertions in *Plasmodium* parasites.

## Abbreviations

NGS: Next generation sequencing; dhfr-ts: Dihydrofolate reductase-thymidylate synthase; eef1aa: Eukaryotic elongation factor 1A alpha; GFP: Green fluorescent protein; UTR: Untranslated regions; ITR: Inverted terminal repeat; SOAP: Short oligonucleotide alignment program; InDel: Insertion and deletion; iPCR: Inverse PCR; SNP: Single nucleotide polymorphism.

## Competing interests

The authors declare that they have no competing interests.

## Authors’ contributions

WP, DZ and YC conceived the study. YC, BR, DLW and ML carried out the laboratory experiments. YC, BC and WP contributed to the data analysis and discussion. YC, DLW, BC, DZ and WP wrote the paper. WP supervised the study. All authors have read and approved the final manuscript.

## Supplementary Material

Additional file 1 **All *piggyBac *insertion sites in the *Plasmodium berghei *genome identified by the ‘BLAST method’, the ‘SOAP method’ and the inverse PCR method, along with results of the half-nested PCR verification and descriptions of each *piggyBac *insertion.** *; The sites outside the brackets were identified by the ‘BLAST method’ or the inverse PCR method, while the sites inside the brackets were identified by the ‘SOAP method’. Each of the insertion sites identified only by the ‘SOAP method’ was positioned in a genomic location with a limited range (see Table 3). Click here for file

Additional file 2 **All the SNPs (n = 1516) in 14 *Plasmodium berghei *chromosomes.** The 95 homozygous SNPs were labelled with ‘^△^’. The 120 of heterozygous SNPs that were considered to be the truly heterozygous SNPs were labelled with ‘*’, which met the criteria that the ratio of the reads supporting the detected base to the reads supporting the reference base was ≧1 and the reads supporting the detected base were ≧10. The 524 of heterozygous SNPs were marked as ‘potential false positive’, as they met the criteria that the ratio of reads supporting the detected base to reads supporting the reference base was <0.5 and the reads supporting the detected base was <10. Click here for file
